# Host Transcriptional Response of *Sclerotinia sclerotiorum* Induced by the Mycoparasite *Coniothyrium minitans*

**DOI:** 10.3389/fmicb.2020.00183

**Published:** 2020-02-11

**Authors:** Huizhang Zhao, Ting Zhou, Jiatao Xie, Jiasen Cheng, Daohong Jiang, Yanping Fu

**Affiliations:** ^1^State Key Laboratory of Agricultural Microbiology, Huazhong Agricultural University, Wuhan, China; ^2^Hubei Key Laboratory of Plant Pathology, College of Plant Science and Technology, Huazhong Agricultural University, Wuhan, China

**Keywords:** *Sclerotinia sclerotiorum*, *Coniothyrium minitans*, shikimate pathway, effector, necrosis- and ethylene-inducing peptide 1, mycoparasitism

## Abstract

Mycoparasite *Coniothyrium minitans* parasitizes specifically the mycelia or sclerotia of *Sclerotinia sclerotiorum*, a worldwidely spread plant fungal pathogen causing serious diseases on crops. The interaction of *C. minitans* with *S. sclerotiorum* remains reciprocal and complex and little is known, especially on the side of the host (*S. sclerotiorum*). In this study, the early transcriptional response of *S. sclerotiorum* to the mycoparasitism by *C. minitans* was explored and the differentially expressed genes (DEGs) were analyzed. Based on GO ontology, KEGG pathway and fungal categories database, 887 up-regulated DEGs were enriched in the growth related function (i.e., rRNA processing, ribosome biogenesis, binding and transport), while the 546 down-regulated DEGs were enriched in the stress-related functions (i.e., oxidoreductase, response to stress and heat and the chorismate biosynthetic process). The expression of shikimate pathway and the biosynthesis of phenylalanine involving genes was significantly suppressed. Furthermore, 581 unenriched DEGs were explored in the parasitizing process and were mapped on the Pfam domains of redox enzymes, Alpha/Beta hydrolase, haloacid dehalogenase, and other universal conserved domain containing proteins. Thirty-two DEGs encoding candidate effectors, with 16 up-regulated and 16 down-regulated, were observed with diverse function. *SS1G_11912* (encoding SsNEP2) was significantly up-regulated and may function in the parasitism. The involving of the shikimate pathway of phenylalanine biosynthesis and effector candidates were discussed. The results provide a basal understand on the interaction of *S. sclerotiorum* and *C. minitans*.

## Introduction

*Sclerotinia sclerotiorum* is a worldwide distributed destructive plant fungal pathogen, attacks more than 400 plant species (Boland and Hall, 1994) and causes huge economic losses (Schwartz and Singh, 2013). *Coniothyrium minitans*, a mycoparasitic fungus specific to some species in *Sclerotinia* (Campbell, 1947; Boland and Hall, 1994), parasitizes hypha and sclerotia of *S. sclerotiorum* efficiently and reduces the sclerotia inoculated in the soil (Campbell, 1947; Huang, 1977; Trutmann et al., 1980; Tu, 1984; Jiang et al., 1996; Li et al., 2006; Whipps et al., 2007; Shukunami et al., 2016). Therefore, *C. minitans* has aroused great interests for its biological control potential and has been developed as commercial biological control agents in many countries, including Germany, Russia, Spain, etc. to control diseases caused by *S. sclerotiorum* and *S. minor* in the field and greenhouses (Budge and Whipps, 1991; Budge et al., 1995; Öhberg and Bång, 2010; Melo et al., 2011; Zeng W. et al., 2012; Kamal et al., 2016; Elsheshtawi et al., 2017). In China, a production certificate was given in 2018 to *C. minitans* strain ZS-1SB, aiming to control stem rot of rapeseed caused by *S. sclerotiorum*.

*Coniothyrium minitans* synthesizes antifungal substances (AFSs) to inhibit the growth of *S. sclerotiorum* (McQuilken et al., 2002; Yang et al., 2007), which could be an important mechanism to control the diseases caused by *S. sclerotiorum.* As a mycoparasite, mycoparasitism is crucial for the biocontrol activity of *C. minitans* (Campbell, 1947; Trutmann et al., 1982). Enzymatic hydrolysis and mechanical pressure were considered two key factors for penetrating *S. sclerotiorum* by *C. minitans* (Jones et al., 1974; Phillips and Price, 1983; Huang and Kokko, 1988). Genes encoding components of MAP kinase cascade (Zeng F. et al., 2012; Wei et al., 2013), NADPH oxidase (Wei et al., 2016), oxalate decarboxylase (Zeng et al., 2014), peroxisome (Wu, 2006; Guo, 2008; Wei et al., 2013), heat shock factors (Hamid et al., 2013), and a transcription factor CmMR1 (Luo et al., 2018) were identified to be involved in the mycoparasitism of *C. minitans*. The interaction of *C. minitans* with *S. sclerotiorum* is reciprocal and complex, though some researches have been reported, the mechanism underling is far more undiscovered, especially on the host side *S. sclerotiorum.*

Similar to the interaction system of pathogens and plants, the defense system of *S. sclerotiorum* would be activated by parasitizing of *C. minitans*. In order to clarify the response of *S. sclerotiorum* to *C. minitans*, in this study, the transcriptome of *S. sclerotiorum* parasitized by *C. minitans* was sequenced and the genes response to parasitism by *C. minitans* were analyzed. Based on our research, some clues on the interaction of *C. minitans* with *S. sclerotiorum* on the host side would be provided and the understand on the interaction would be deeply enhanced.

## Results

### Identification of Differentially Expressed Genes (DEGs) of *S. sclerotiorum* Induced by *C. minitans*

In order to identify the gene expression profile of *S. sclerotiorum*, mycelial samples of *S. sclerotiorum* were collected 0, 4, and 12 h after co-cultured with hypha of *C. minitans* and three transcripted RNA libraries named SsCm0h, SsCm4h, and SsCm12h were constructed accordingly. More than 9.92 million clean reads were generated from each library, with approximately 5.74, 4.56, and 4.76 million were mapped to the genome of *S. sclerotiorum* in each library. The unique match of clean reads were 5.53, 4.42, and 4.53 million for each library, respectively, reflecting a high quality of the effective sequencing data (Supplementary Table S1). The mapped metadata files were uploaded into sequence read archive (SRA) with SRA accessions SRR10436181, SRR10436182, SRR10436183 for SsCm0h, SsCm4h, and SsCm12h, respectively.

**FIGURE 1:**
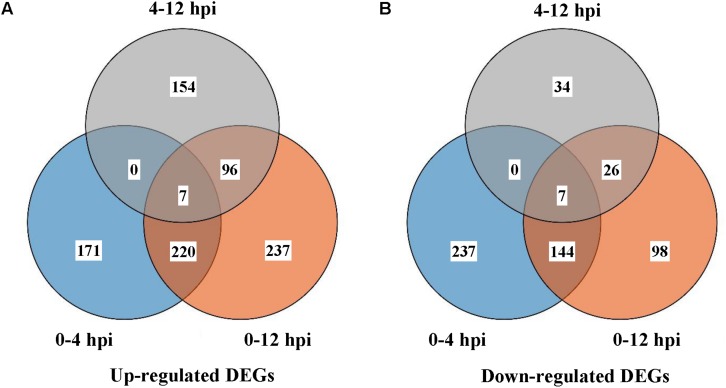
Differentially expressed genes (DEGs) in *S. sclerotiorum* 0, 4, or 12 h after interacting with *C. minitans*. **(A)** Up-regulated DEGs of different comparison groups; **(B)** Down-regulated DEGs. Comparison groups were conducted between the different interaction time points. SsCm0h-vs-SsCm4h, SsCm0h-vs-SsCm12h and SsCm4h-vs-SsCm12h, the comparison groups of co-culture 4 h was versus to 0, 12 versus to 0 and 12 h compared to 4 h, respectively.

11125, 10843, and 11083 expressed genes in *S. sclerotiorum* were detected in libraries of SsCm0h, SsCm4h, and SsCm12h, respectively. The gene expression of *S. sclerotiorum* was compared to each parasitizing stage and a total of 1368 DEGs were detected during all the early stages of mycoparasitism process. During 0–4 hpi, 171 stage-specific DEGs were up-regulated and 237 were down-regulated; 237 up-regulated DEGs and 98 down-regulated DEGs were identified during 0–12 hpi; 154 up-regulated DEGs and 34 down-regulated DEGs were identified during 4–12 hpi; 227 up-regulated DEGs and 151 down-regulated DEGs were shared at the two stages of 4 or 12 hpi comparing to 0 hpi (Figure 1).

### Functional Classification of DEGs in *S. sclerotiorum*

**FIGURE 2:**
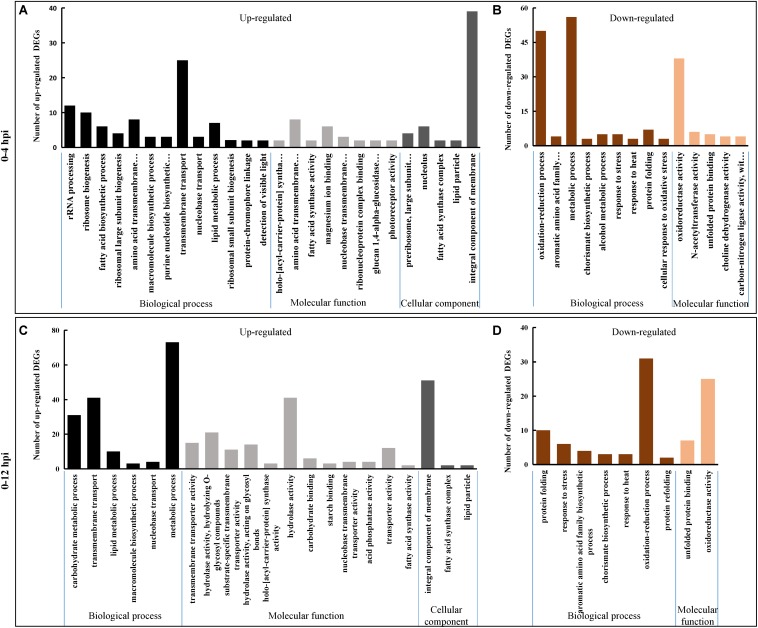
Gene Ontology (GO) classification of *S. sclerotiorum* DEGs. Genes were annotated in three categories: biological process (BP), molecular function (MF) and cellular component (CC). Y-axis represents the DEG number of a specific category. **(A,B)** Represent *S. sclerotiorum* up-regulated DEGs and down-regulated DEGs in 0–4 hpi, respectively. **(C,D)** Represent *S. sclerotiorum* up-regulated DEGs and down-regulated DEGs in 0–12 hpi, respectively. The detailed information was shown in Supplementary Table S2.

During 0–4 hpi, for the up-regulated DEGs, GO terms related to microbial growth including ‘rRNA processing (GO: 0006364),’ ‘transmembrane transport (GO: 0055085),’ and ‘macromolecule biosynthetic process (GO:0009059)’ were enriched (Figure 2A and Supplementary Table S2). While the down-regulated DEGs were enriched into the terms ‘oxidation-reduction process (GO: 0055114),’ ‘aromatic amino acid family biosynthetic process (GO: 0009073),’ ‘chorismate biosynthetic process (GO: 0009423),’ ‘protein folding (GO: 0006457),’ ‘response to stress (GO: 0006950),’ and ‘response to heat (GO: 0009408)’ (Figure 2B and Supplementary Table S2). The same catagories could be detected during 0–12 hpi for the down-regulated DEGs, while the term ‘carbohydrate metabolic process (GO:0005975)’ involved in the term ‘hydrolase activity (GO: 0016787)’ was also enriched for the up-regulated DEGs (Figures 2C,D and Supplementary Table S2).

**FIGURE 3:**
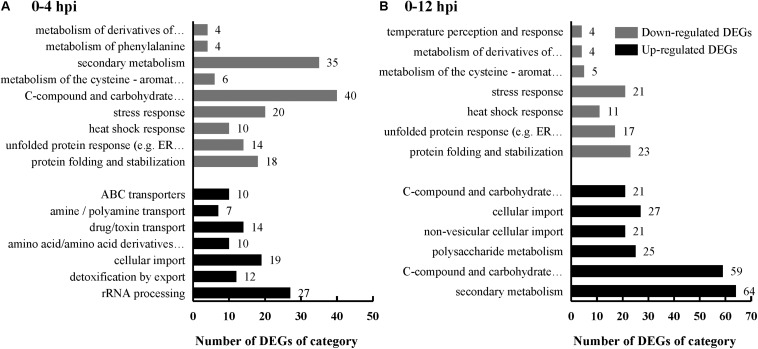
FunCat category enrichment of *S. sclerotiorum* DEGs induced by *C. minitans*. Genes were analysized according to the web-based FungiFun2 database against genome of *S. sclerotiorum*. X-axis represents the DEG number of a functional category. Black bars represent the up-regulated DEGs and gray bars represent the down-regulated DEGs. **(A)** *S. sclerotiorum* DEGs in 0–4 hpi. **(B)** *S. sclerotiorum* DEGs in 0–12 hpi. The detailed information was shown in Supplementary Table S3.

*Sclerotinia sclerotiorum* DEGs were mapped onto Fungi Category (FunCat) database to identify categories that were significantly (*p* ≤ 0.05) regulated when parasitized by *C. minitans*. FunCat category “rRNA processing (FCID: 11.04.01),” “cellular import (FCID: 20.09.18),” “non-vesicular cellular import (FCID: 20.09.18.07),” “C-compound and carbohydrate transport (FCID: 20.01.03),” and “polysaccharide metabolism (FCID: 01.05.03)” were enriched among the up-regulated DEGs (Figure 3 and Supplementary Table S3). While “protein folding and stabilization (FCID: 14.01),” “unfolded protein response (FCID: 32.01.07),” “heat shock response (FCID: 32.01.05),” “stress response (FCID: 32.01),” “metabolism of the cysteine-aromatic group (FCID: 01.01.09),” “metabolism of phenylalanine (FCID: 01.01.09.04),” and “metabolism of derivatives of dehydroquinic acid, shikimic acid, and chorismic acid (FCID: 01.20.15)” were significantly enriched among the down-regulated DEGs during the early parasitized process of *S. sclerotiorum* by *C. minitans* (Figure 3 and Supplementary Table S3).

Based on the KEGG pathway assessment, the “metabolism pathway” was the most significantly enriched pathway (Supplementary Table S4). The fatty acid metabolism (map 01212) and biosynthesis (map 00061), biosynthesis of unsaturated fatty acids (map 01040), steroid biosynthesis (map 00100), starch and sucrose metabolism (map 00500) and propanoate metabolism (map 00640) and ribosome biogenesis in eukaryotes (map 03008) were significantly enriched among the up-regulated DEGs; while the pathway phenylalanine, tyrosine, and tryptophan biosynthesis (map 00400) was enriched pathway among the down-regulated DEGs.

Altogether, functional enrichment of the DEGs showed that physiologically related genes in *S. sclerotiorum* induced by *C. minitans* were enriched and up-regulated during the early stages, while stress-stimulus related genes were enriched and down-regulated.

### The Shikimate Pathway Was Suppressed in *S. sclerotiorum* Challenged by *C. minitans*

**FIGURE 4:**
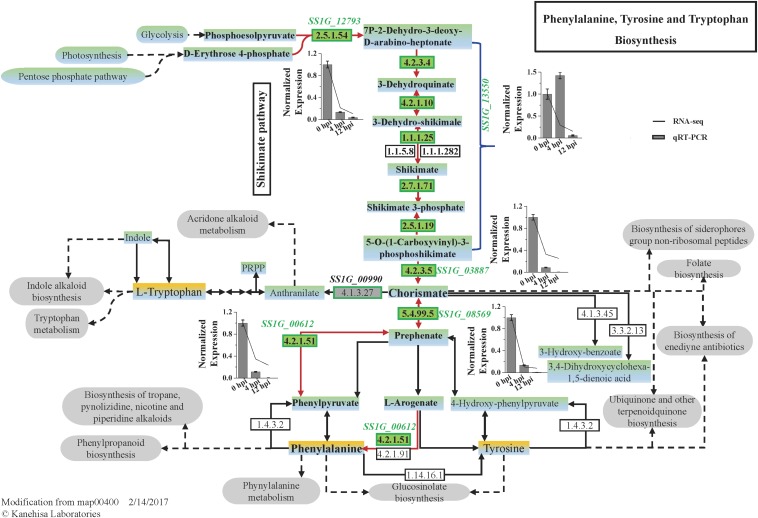
The pathway map of Phenylalanine, Tyrosine, and Tryptophan Biosynthesis (map 00400) predicted in *S. sclerotiorum* and the DEGs enriched in the pathway induced by *C. minitans*. The italics are the genes putatively involved in the phenylalanine, tyrosine, and tryptophan biosynthesis process. The numbers in the green block are the EC (Enzyme Commission) numbers in enzyme nomenclature databasehttps://enzyme.expasy.org/ and were predicted to be encoded by genes presented near the green box. During the early interactional stages of *S. sclerotiorum* induced by *C. minitans*, the gene expression identified in the biosynthesis process was detected in the interactional transcriptome and confirmed by using qRT-PCR. In bar charts, the lines show the relative expression from RNA_seq and the bars represent the data from qRT-PCR. The gene expression of 0 hpi was set as 1 and the error bar was calculated with three replications. The KEGG pathway of map00400 was download from https://www.kegg.jp/ and the gray oval frames are predicted to be the related pathways of the phenylalanine, tyrosine, and tryptophan biosynthesis process.

The expression of five DEGs involved in the biosynthesis of phenylalanine was significantly suppressed (Figure 4). Gene *SS1G_13550* is deduced to encode a multifunctional protein, including dehydroquinate synthase (EC 4.2.3.4) (DHQS), 3-dehydroquinate dehydratase (EC 4.2.1.10) (DHQD), shikimate dehydrogenase (E.C. 1.1.1.25) (SKDH), shikimate kinase (EC 2.7.1.71) (SHK) and 5-O-(1-Carboxyvinyl)-3-phosphoshikimate synthase (EC 2.5.1.19) (EPSPS), catalyzing the five steps from DAHP (7P-2-Dehydro-3-deoxy-D-arabino-heptonate) to EPSP (5-Enolpyruvylshikimate 3-phosphate) of the shikimate pathway. The first key step is catalyzed by *SS1G_12793* encoding DAHP synthase (E.C. 2.5.1.54) and the final step to form chorismate was catalyzed by the enzyme chorismate synthase (E.C. 4.2.3.5) encoded by *SS1G_03887*. All these three genes were significantly down-regulated during the early mycoparasitism stages (Figure 4). A gene *SS1G_08569* encoding chorismate mutase (E.C. 5.4.99.5), catalyzing the conversion of chorismate to prephenate, was significantly down-regulated at 4 hpi (log_2_Ratio = −3.29) and 12 hpi (log_2_Ratio = −3.64) than 0 hpi (Figure 4). Followed closely, the step converting prephenate to phenylpyruvate is catalyzed by prephenate dehydratase, which was predicted to be encoded by *SS1G_00612* in *S. sclerotiorum*, and the gene expression was suppressed at the early mycoparasitism stages by *C. minitans* (Figure 4). The results showed that the shikimate pathway might play an important role in the defense of *S. sclerotiorum* to *C. minitans*.

### Expression of Effector Encoding Genes Was Significantly Regulated in *S. sclerotiorum* Challenged by *C. minitans*

**FIGURE 5:**
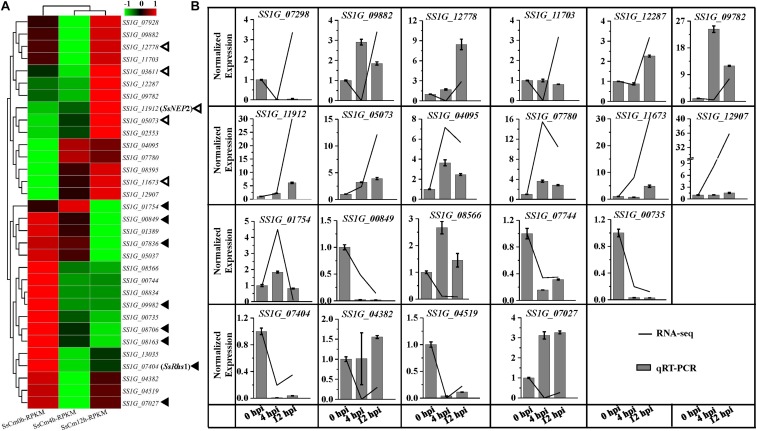
Comparsion of RNA-seq and qRT-PCR results for the relative expression of predicted effector encoding genes at 0, 4, 12 hpi of *S. sclerotiorum* induced by *C. minitans*. **(A)** Gene expression of the 32 detected effector encoding gene of *S. sclerotiorum* during the early parasitized stages of *S. sclerotiorum* using RNA sequencing. Hollow triangles represent the five DEGs with similar gene patterns to pathogenic process; the solid black triangles indicate the eight DEGs with different patterns in parasitism process from pathogenic process. **(B)** Verification of 12 significantly up-regulated and 8 down-regulated effector encoding genes of *S. sclerotiorum* using qRT-PCR. The gene changes in transcript abundance of RNA-seq were normalized with RPKM, and for the qRT-PCR, all the data were normalized against the expression of the β-tubulin gene and the gene expression at 0 hpi was set control as 1. The related primer pairs were listed in Supplementary Table S7.

There are 695 secretory proteins identified in the genome of *S. sclerotiorum* strain 1980, among which 304 proteins were predicted as conventional effector candidates. During the early stages in *S. sclerotiorum* challenged by *C. minitans*, 129 DEGs encoded secretory proteins, accounting for 9.4% of total detected DEGs (Supplementary Table S5). Totally 32 DEGs encoded candidate effectors, with 16 up-regulated and the other 16 down-regulated (Figure 5A). Among these genes, *SS1G_11912*, encoding a predicted effector with a domain of necrosis-inducing protein 1 (NPP1), was significantly up-regulated (4.91-fold of 12 to 0 hpi) at the early stages of mycoparasitism (Figure 5B).

### Analysis of Unclassified DEGs in *S. sclerotiorum*

**FIGURE 6:**
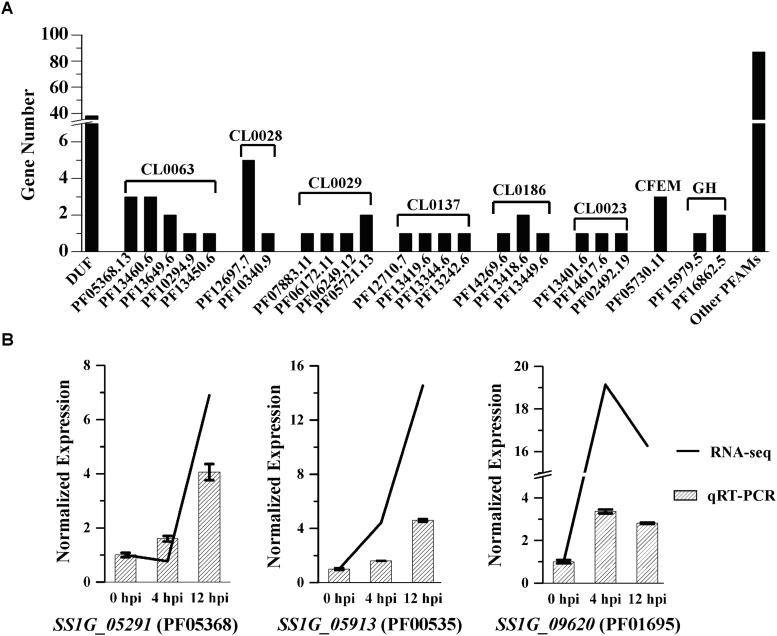
Protein family (Pfam) annotation of the unclassified DEGs in *S. sclerotiorum* induced by *C. minitans*. **(A)** Gene count annotated into the Pfam family of the unclassified DEGs. DUF, protein of unknown function; PFxxx, protein family list in the Pfam database; CLxxx, the clan family based on the Pfam databse; CFEM, a fungal specific cysteine rich domain; GH, domain of glycoside hydrolase. **(B)** Valuation of the three high regulated DEGs of *SS1G_05291*, *SS1G_05913*, and *SS1G_09620* based on qRT-PCR during the early interaction stages of 0, 4, and 12 hpi in *S. sclerotiorum* induced by *C. minitans*. The primer pairs of *SS1G_05291*, *SS1G_05913*, and *SS1G_09620* were detail listed in Supplementary Table S7.

During the early mycoparasitic process, 580 DEGs in *S. sclerotiorum* could not be classified into any terms of GO ontology, KEGG pathway or FunCat category. Almost all the unclassified DEGs were hypothetical proteins without predicted function based on blast against the non-redundant protein (Nr) database (Supplementary Table S6). Proteins encoded by 163 DEGs have been annotated with functional domains against Pfam database, including 38 proteins with domain of unknown function, 125 proteins with similar functional domains reported in other microorganisms (Supplementary Table S6). The top five clan families were NADP_Rossmann (CL0063), AB_hydrolase (CL0028), Cupin (CL0029), HAD (CL0137), and Beta_propeller (CL0186) (Figure 6A and Supplementary Table S6). Some unclassified DEGs were identified with domains related to the function of redox enzymes, Alpha/Beta hydrolase, haloacid dehalogenase, and some with universal conserved-domains (i.e., CL0029 and CL0186) (Murzin, 1992; Ollis et al., 1992; Koonin and Tatusov, 1994; Dunwell, 1998; Dunwell et al., 2004). *SS1G_05913*, *SS1G_09620*, and *SS1G_05291* encoding proteins with domain of Glycosyltransferase family 2 (GT2) (PF00535), DJ-1/PfpI family (PF01965) and NmrA-like family (PF05368) respectively, were highly expressed and significantly up-regulated (log_2_Ratio > 3.8 and FDR ≤ 0.001) at 12 hpi induced by *C. minitans* and the express were further confirmed by qRT-PCR (Figure 6B). Combined with the functional analysis of the DEGs, the results hint an intricate response of *S. sclerotiorum* to the parasitism by *C. minitans*.

### Expression of *NPP*1 Related Genes in *S. sclerotiorum* Challenged by *C. minitans*

In order to discover the function of NPP1 related genes in the interaction of *S. sclerotiorum* and *C. minitans*, NPP1 homologs were analyzed in the genome of *S. sclerotiorum*, its parasitic fungus *C. minitans* and a pathogenic fungus *Botrytis cinerea* B05.10. Two homologs existed in each of the three genome. In *S. sclerotiorum, SsNEP*2 was approximately induced 4.9-fold at 12 hpi compared with the initial contacting stage of 0 hpi with *C. minitans* checked by RNA-seq and qRT-PCR (Figure 2), while *SsNEP*1 kept at a relatively low level during the parasitism process (Supplementary Figure S1a). In *B. cinerea*, the most closely phylogenetic fungus of *S. sclerotiorum* and a non-host fungus of *C. minitans*, expression of *BcNEP*1 and *BcNEP*2 were not influenced by contacting with *C. minitans* (Supplementary Figures S1b,c). The results suggest that *SsNEP*2 may be involved in the interaction of *S. sclerotiorum* and *C. minitans.*

## Discussion

It is generally accepted that the pathogenic microbes induce host plant responses and inhibit the immune stress-related gene expression (Jones and Dangl, 2006; Irieda et al., 2019). Similarly, as a host, *S. sclerotiorum* may response to the parasitizing of parasite *C. minitans.* In this research, the transcriptional responses of *S. sclerotiorum* 4 and 12 h after contacting with *C. minitans* were monitored by RNA-seq.

Shikimate pathway presents in plants, bacteria, fungi, and certain protozoans including apicomplexan parasites, while is absent in animals (Keeling et al., 1999; Roberts et al., 2002). Shikimate pathway is the common aromatic biosynthetic pathway that involves seven enzymatic steps for the conversion of phosphoenolpyruvate and erythrose 4-phosphate to chorismate, providing the precursor of the three aromatic amino acids (phenylalanine, tyrosine, and tryptophan) and many aromatic secondary metabolites (Herrmann and Weaver, 1999; Macheroux et al., 1999; Derrer et al., 2013; Mir et al., 2015). Increased enzyme activity of the shikimate pathway in plants enhances the resistance to pathogens (Roberts et al., 1998; Guillermo and Miller, 2004; Rippert et al., 2004). The metabolite level of the shikimate pathway was induced in the *Magnaporthe oryzae*-challenged rice roots and the resistance to pathogen infection was increased (Xu et al., 2015). Shikimate dehydrogenase (SKDH) is an active site of the multifunction arom protein and is considered to be the most promising component related to the plant resistance to *S. sclerotiorum* (Enferadi et al., 2011). Overexpression of barley chorismate mutase 1 (*HvCM*1) or chorismate synthase (*HvCS*) significantly increased the resistance to *Blumeria graminis* penetration in barley, while gene silencing increased susceptibility (Pingsha et al., 2009).

Shikimate pathway is also important for development and pathogenicity in fungi. Seven enzymes participating in the pathway were usually designed as antimicrobial and anti-parasitic drug targets (Roberts et al., 1998; Keeling et al., 1999; Aditya et al., 2012; Tzin et al., 2012; Mir et al., 2015). The chorismate mutase *Cmu*1 secreted by *Ustilago maydis* was a virulence factor and deletion mutants of the gene led to virulence attenuation on maize, with correspondingly up-regulated during biotrophic development of *U. maydis* (Djamei et al., 2011). *SS1G_14320* encoding a chorismate mutase was expressed when cultured on PDB and throughout the infection process and predicted to be an important function in *Brassica napus* infection (Derbyshire et al., 2017). In this research, *SS1G_14320* was also up-regulated, while another chorismate mutase encoding gene *SS1G_08569* was detected down-regulated in *S. sclerotiorum* challenged by *C. minitans*. These may suggest that *S. sclerotiorum* recruits different genes to encode chorismate mutase in the interaction with host plant or *C. minitans*. Five genes encoding enzymes involved in the shikimate pathway and phenylalanine biosynthesis were significantly suppressed in *S. sclerotiorum* at the early stage of mycoparasitism process by *C. minitans*, namely *SS1G_12793, SS1G_13550, SS1G_03887, SS1G_08569*, and *SS1G_00612*. *C. minitans* probably decreases the resistance of *S. sclerotiorum* by inhibiting the shikimate pathway and the phenylalanine biosynthesis to profit its parasitization.

As a typical necrotrophic fungal pathogen, *S. sclerotiorum* is also reported to secrete effectors to manipulate host cells. 363 small secretary proteins with less than 300 amino acids in size were predicted in the genome of *S. sclerotiorum* (Amselem et al., 2011). Using different criteria, 79 and 70 effector candidates with diverse patterns of expression were identified to be involved in a wide range of functions, including chitin binding, proteases and protease inhibitors during the interaction with host plants (Amselem et al., 2011; Guyon et al., 2014; Heard et al., 2015; Derbyshire et al., 2017; Seifbarghi et al., 2017; Westrick et al., 2019). Recently, Westrick et al. (2019) found 57 DEGs encoded putative secreted effectors in *S. sclerotiorum* when infecting *Glycine max.* Out of these genes, 18 were differentially regulated at the late infection stage (96 hpi) comparing to the early stages (average of 24 and 48 hpi) (Westrick et al., 2019). In this study, 13 pathogenic effector candidate coding genes of *S. sclerotiorum in planta* were also expressed when challenged by *C. minitans* with five up-regulated and the other eight down-regulated. For example, *SS1G_03611*, encoding a CFEM domain with proposed roles in fungal pathogenesis or conserved fungal effector domains, was induced during *S. sclerotiorum* infection on plants (Kulkarni et al., 2003; Heard et al., 2015) and was also up-regulated (1.84-fold) when challenged by *C. minitans*. Similar expression patterns were also observed for other four genes, *SS1G_11912* (SsNEP2), *SS1G_12778* (encoding a necrosis-inducing effector), *SS1G_05073* (encoding a phospholipase C-like enzyme), and *SS1G_11673* (encoding a putative pathogenic effector) (Bashi et al., 2010; Guyon et al., 2014; Derbyshire et al., 2017; Seifbarghi et al., 2017; Westrick et al., 2019). Eight pathogenic effector required for virulence on plant host and highly induced on plant were inhibited or undetectable during the early mycoparasitic stages of *S. sclerotiorum* by *C. minitans*, including *SS1G_07404* (Ss-Rhs1, rearrangement hotspot repeat 1), *SS1G_01754*, *SS1G_00849*, *SS1G_07836*, *SS1G_09982*, *SS1G_08706*, *SS1G_08163*, and *SS1G_07027* (Guyon et al., 2014; Derbyshire et al., 2017; Seifbarghi et al., 2017; Westrick et al., 2019). Nine effectors of ssv263 (Liang et al., 2013), SsCutA (Bashi et al., 2012), SsPG1 (Bashi et al., 2012), SSITL (Zhu et al., 2013), SsECP6 (Heard et al., 2015), SsCP1 (Yang et al., 2018), SsSSVP1 (Lyu et al., 2016), SsCVNH (Lyu et al., 2015), and SsSm1 (Pan et al., 2018) were confirmed to be required for full virulence on plant host and expression of the encoding genes were up-regulated in plant infection, while the gene expressions did not show any difference during the early parasitism stages. The phenomenon suggests that *S. sclerotiorum* responds in a different manner to plant hosts and to *C. minitans*.

NPP1 is a conversed peptides triggering the transcript accumulation of pathogenesis-related (PR) genes, production of ROS and ethylene, callose apposition, and HR-like cell death in plants (Fellbrich et al., 2002; Qutob et al., 2006; Oome et al., 2014). *Fusarium oxysporum* NEP1 induced rapid structural changes, including the thinning of the cuticle and disruption of chloroplasts in spotted knapweed, dandelion, and Arabidopsis (Keates et al., 2003). *S. sclerotiorum* NEPs caused necrosis on tobacco leaves (Bashi et al., 2010). However, there is no direct proof to support the effects of *SsNep*2 expression on virulence though *SsNep*2 was expressed within 6 h and peaked at 24 h after inoculated on *B. napus* leaves (Bashi et al., 2010). Here we found that *SsNEP*2 was significantly up-regulated in *S. sclerotiorum* challenged by *C. minitans*, while the expression of homolog *BcNEP*2 was relatively stable in the non-host *B. cinerea* challenged by *C. minitans* (Supplementay Figures S1b,c). Therefore, the role of SsNEP2 in the parasitic system of *S. sclerotiorum* and *C. minitans* need further investigation.

*Coniothyrium minitans* has been used as a biological agent to control diseases caused by *S. sclerotiorum* and *S. minor.* The foliar application of *C. minitans* conidia on bean plants during the early bloom to mid-bloom period reduced the proportion of plants infected by an average of 56% (*p* < 0.001) (Huang et al., 2000). Hyphal extension of *S. sclerotiorum* was inhibited by 68% when germinated conidia of *C. minitans* were spread on leaves of oilseed rape (Shi et al., 2004). Approximately 76% decrease of disease lesions was developed when flower petals of *Brassica* spp. were treated with *C. minitans* + *S. sclerotiorum* (Li et al., 2006). When contact with each other on oilseed rape, during parasitizing on the hypha, *C. minitans* may suppress the shikimate pathway in *S. sclerotiorum* and alter effector-like proteins to attenuate the virulence of *S. sclerotiorum*, and finally control the *Sclerotia* stem rot of crops.

In this study, we have only surveyed the transcriptional response of the host *S. sclerotiorum* induced by *C. minitans* during the early mycoparasitic stages. The interaction of *S. sclerotiorum* parasitized by *C. minitans* is a two-way process, and a considerable number of reads mapped to the genome of *C. minitans* were also included in the interactive RNA_seq libraries. The responses of *C. minitans* need further study in order to elucidate the two-way interaction and a great opportunity would be provided to understand the mycoparasitism mechanism.

## Materials and Methods

### Strain and Growth Conditions

*Coniothyrium minitans* strain ZS-1 was used to activate the response of *S. sclerotiorum* strain 1980 or *B. cinerea* strain B05.10 with hyphal contact. All the strains were grown on potato dextrose agar plates (PDA) (BD Biosciences, Franklin Lakes, NJ, United States) at 20°C.

### RNA-seq Preparation and Sequencing

Mycelia of strain 1980 were cultured on the sterile cellophane membrane, which was placed on the PDA plates (Φ = 9 cm); 12 h late, the conidia of *C. minitans* strain ZS-1 were shaken at 20°C in PDB at 150 rpm for 36 h, washed with sterilized water for three times and re-suspended in water to 1.0 × 10^6^ conidia mL^–1^. The conidial suspension was spread on the 48-h-old colony (1 mL for each plate) of *S. sclerotiorum*. The mycelial mixtures were sampled at three interactional points of 0 hpi (immediately after coating), 4 hpi (co-culture for 4 h) and 12 hpi (co-culture for 12 h), and total RNA was extracted using RNA reagent (NewBio Industry, Tianjin, China) following the instructions. mRNA was enriched by using the oligo(dT) magnetic beads for the first strand cDNA synthesization by random hexamer-primer. Then buffer, dNTPs, RNase H, and DNA polymerase I were added to sythsize the second strand and the double strand cDNA was purified with QiaQuick PCR extraction kit (Qiagen, Mainz, Germany). Finally, fragments from the double strand cDNA were ligated with sequencing adaptors to construct the sequencing library. The library products were sequenced via an Illumina HiSeq 2000 at 49 bps of single-end read at BGI. One library was constructed for each interactional point and qRT-PCR was used to confirming the interactional transcriptome.

### Analysis of Differentially Expressed Genes (DEGs) at Different Interaction Time Points

Adaptors, reads with more than 10% unknown bases and low-quality reads (quality value ≤ 5 of a read) were removed from the raw reads to obtain the clean reads. The clean reads were mapped to the genome of *S. sclerotiorum* using SOAP aligner/soap2 (Li et al., 2009). Mismatches of no more than two bases were allowed in the alignment. The gene expression level was calculated using the RPKM method (reads per kb per million reads) (Mortazavi et al., 2008).

A method described in “The significance of digital gene expression profiles” (Audic and Claverie, 1997) was used to screen the DEGs. We used FDR (False Discovery Rate) ≤ 0.001 and the absolute value of log_2_| ratio| ≥ 1.5 as the threshold to judge the significance of gene expression differences.

Three groups of RPKM-based gene expression data were obtained from the three RNA-seq libraries based on the genome of *S. sclerotiorum*. SsCm0h, SsCm4h, and SsCm12h represented the gene expression of *S. sclerotiorum* at 0, 4, and 12 h mycoparasitism stages induced by *C. minitans*, respectively. Gene expression data of *S. sclerotiorum* in SsCm0h, SsCm4h, and SsCm12h with clean reads ≥ 10 in one of the time points were retained. Based on the gene RPKM value data, a series of comparison groups were conducted to analyze the mycoparasitism-related genes among the early interaction stages. We examined the DEGs at different interaction stages, and three comparisons were conducted: 0–4 hpi, gene expression of *S. sclerotiorum* in CmSs4h versus CmSs0h; 0–12 hpi, CmSs12h versus CmSs0h; and 4–12 hpi, CmSs12h versus CmSs4h.

### Functional Enrichment Analyses of DEGs

Differentially expressed genes in *S. sclerotiorum* were enriched based on the functional categorization of *S. sclerotiorum* in FungiFun2^1^ online (Priebe et al., 2015). The significant level of the three analyses of GO ontology (GO), KEGG pathway (KEGG) and Fungi Categories (FungiFun) were cut at 0.05 with Fisher’s exact test. The protein families (Pfam) annotations were conducted using HMMER software (version 3.2.1) with the hmmscan algorithm based on the database of Pfam (version 32.0) at default parameters (Eddy, 2011; El-Gebali et al., 2019).

### Effectors Prediction and Annotation

We used complete genome and predicted proteomes of *S. sclerotiorum* strain 1980 downloaded from the NCBI (Amselem et al., 2011) to speculate the putative effectors. The presence of secretion signals was predicted with SignalP v.4 (Nielsen, 2017), transmembrane helices and GPI anchor sequence were predicted with TMHMM (Krogh et al., 2001) and GPIsom (Fankhauser and Maser, 2005), respectively. Effectors were set as under 300 amino-acids in length. For the identification of genes expressed in *S. sclerotiorum* challenged by *C. minitans*, RNA-seq data for gene induction fold at 0, 4, and 12 hpi were used. The predicted proteins were annotated using Blast2GO (Conesa et al., 2005) and PFAM^2^. Pfam domains were annotated using HMMER3 searches against the PFAM 32.0 database e-value < 0.01 (El-Gebali et al., 2019).

### Quantitative RT-PCR (qRT-PCR)

The RNA samples were prepared as described in Section “RNA-seq Preparation and Sequencing” and additional treatment with *B. cinerea* instead of *S. sclerotiorum* was set as the non-host control set. The level of gene expression was determined on a Bio-Rad CFX Real Real-time System (Bio-Rad, Berkeley, CA, United States). The cDNA was synthesized using oligo(d)T primer in the EasyScript One-Step gDNA Removal and cDNA Synthesis SuperMix Kit (TransGen biotechnology, Beijing, China). Each PCR reaction contained 7.5 μL of 2 × iTaq Universal SYBR Green Supermix (Bio-Rad, Berkeley, CA, United States), 0.2 μL of cDNA template, 0.3 μL of each primer and 6.7 μL of ddH_2_O. The program was as follows: 95°C for 2 min, followed by 42 cycles of 95°C for 15 s, 57°C for 15 s and 72°C for 15 s, and a cycle with 0.5°C per second from 65 to 95°C to remove the influence of primer dimer. Total cDNA abundance in the samples was standardized against the *S. sclerotiorum* β-tubulin gene. The primers used to obtain an amplicon of approximately 100–150 bp from each target gene are listed in Supplementary Table S4. All samples were amplified in triplicate. Three independent repeats of the experiment were performed in the same way.

### Statistical Analysis

The significant value of the differences in our analyses was evaluated with ANOVA program in the software SAS9.2 at the significant level of *p* = 0.01.

## Conclusion

Although the biocontrol mechanism of *C. minitans* to *S. sclerotiorum* has been studied since *C. minitans* was first reported in Campbell (1947), knowledge about the mycoparasitic process is still at its infancy. In this paper, based on the transcriptome data, we identified “host fungus” response signatures in *S. sclerotiorum* (the host) induced by *C. minitans* (the mycoparasite) that the up-regulated transcripts were enriched mainly on function of lifestyle, while down-regulated transcripts were enriched on responses to stimulus. Genes involved in the shikimate pathway were inhibited and effector encoding genes were regulated. Collectively, our data indicate that *S. sclerotiorum* deployed different genes or different expression patterns to infect plants or respond to the parasitism of *C. minitans*.

## Data Availability Statement

The datasets generated for this study can be found in the mapped metadata files were uploaded into sequence read archive (SRA) with SRA accessions SRR10436181, SRR10436182, SRR10436183 of SsCm0h, SsCm4h, and SsCm12h, respectively.

## Author Contributions

DJ and YF designed the research. HZ and YF wrote the manuscript. HZ and TZ executed the experiments. HZ, JC, and JX performed the data and bioinformatics analyses. All authors read and approved the final manuscript.

## Conflict of Interest

The authors declare that the research was conducted in the absence of any commercial or financial relationships that could be construed as a potential conflict of interest.

## Footnotes

1 https://sbi.hki-jena.de/fungifun/fungifun.php

2 http://pfam.xfam.org/

## Supplementary Material

FIGURE S1 The relative expression of NEP related genes of *S. sclerotiorum* and *B. cinerea.* Mycelia of strain 1980 or B05.10 were cultured on sterile cellophane membrane for 12 h and covered with conidial suspension (1.0 × 10^6^ conidia mL^–1^) of *C. minitans.* The mycelial mixtures were sampled at 0 hpi (immediately after coating), 4 hpi (co-culture for 4 h), or 12 hpi (co-culture for 12 h), and RNA was extracted for qRT-PCR. The gene changes in transcript abundance were normalized with RPKM. For qRT-PCR, the gene expression was normalized against that of β-tubulin gene in *S. scleritorum*. The qRT-PCR valued the gene expression at 0 hpi set as 1. **(a)** *SsNEP*1; **(b)** *BcNEP*1; **(c)** *BcNEP*2. The primers of qRT-PCR for *SsNEP*1, *BcNEP*1, and *BcNEP*2 were listed in Supplementary Table S7.

TABLE S1 The statistics of RNA_seq mapped to the genome of *Sclerotinia sclerotiorum*. The clean reads with mismatches of no more than two bases allowed in the alignment were mapped to the genome of *S. sclerotiorum* using SOAP aligner/soap2.

TABLE S2 GO categories analysis of the DEGs of *S. sclerotiorum* during mycoparasitism process induced by *C. minitans* (*p* ≤ 0.05). The GO classification was conducted on the online website of FungiFun (https://sbi.hki-jena.de/fungifun/fungifun.php) based on GO ontology database of the fungi species of *S. sclerotiorum*.

TABLE S3 Enrichment of FunCat categories of the DEGs in *S. sclerotiorum* (*p* ≤ 0.05). The classification of FunCat categories was conducted on the online website of FungiFun (https://sbi.hki-jena.de/fungifun/fungifun.php) based on FunCat classification database of the fungi species of *S. sclerotiorum*.

TABLE S4 The significantly enriched KEGG pathway of the DEGs of *S. sclerotiorum* during the early mycoparasitism stages (*p* ≤ 0.05). The KEGG pathway analysis was conducted on the online website of FungiFun (https://sbi.hki-jena.de/fungifun/fungifun.php) based on KEGG pathway classification database of the fungi species of *S. sclerotiorum*.

TABLE S5 The secretory proteins in *S. sclerotiroum* and the related DEGs in *S. sclerotiorum* induced by *C. minitans*.

TABLE S6 Gene list of the unclassified DEGs in *S. sclerotiorum* induced by *C. minitans*.

TABLE S7 Primers involved this research.
