# Supplementary figures and images for: Host Transcriptional Response of Sclerotinia sclerotiorum Induced by the Mycoparasite Coniothyrium minitans
Source: Front Microbiol. 2020 Feb 11;11:183. doi: 10.3389/fmicb.2020.00183 (PMC7026392; doi:10.3389/fmicb.2020.00183)

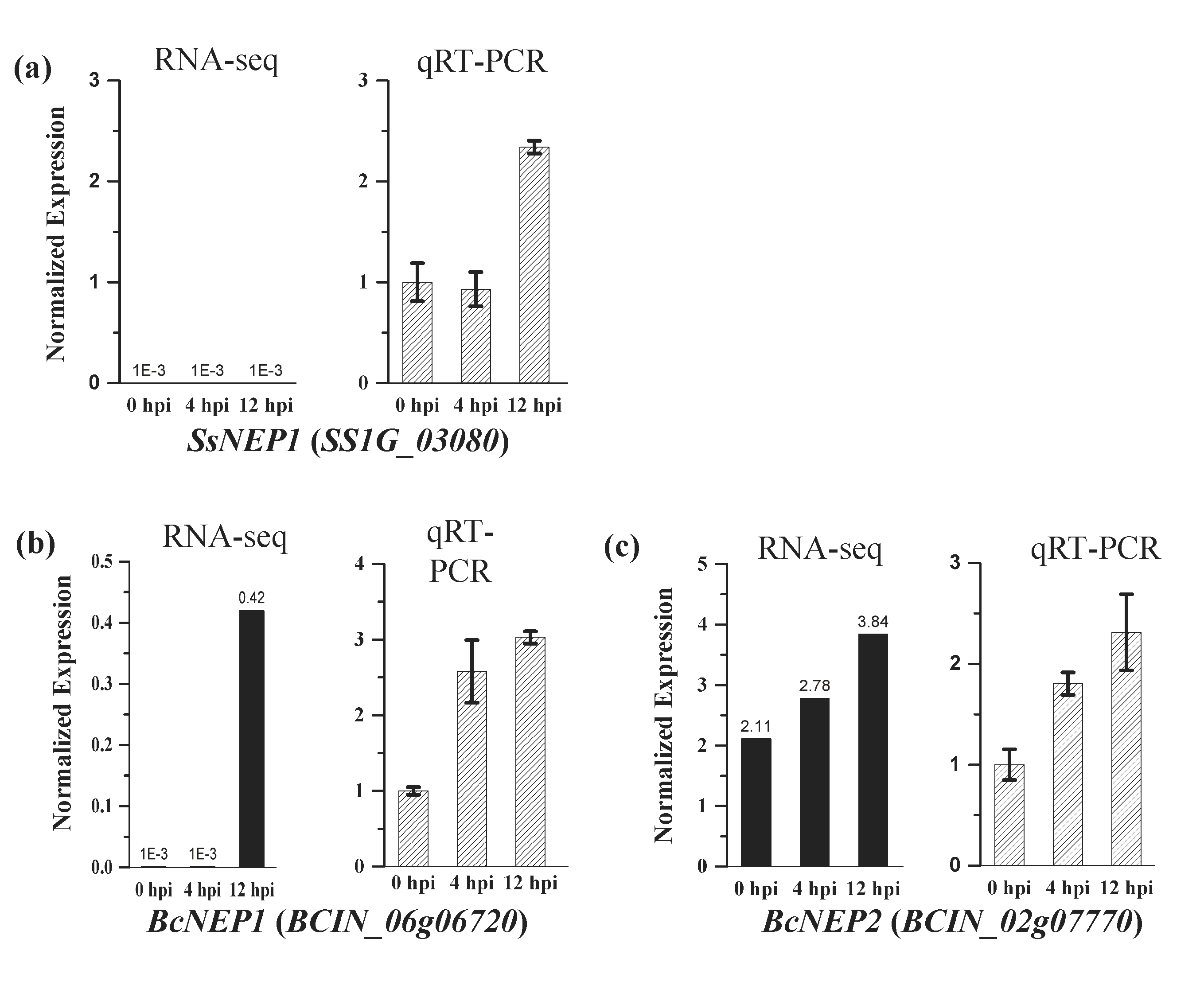

Supplement: FIGURE S1 — The relative expression of NEP related genes of S. sclerotiorum and B. cinerea. Mycelia of strain 1980 or B05.10 were cultured on sterile cellophane membrane for 12 h and covered with conidial suspension (1.0 × 10^6^ conidia mL–1) of C. minitans. The mycelial mixtures were sampled at 0 hpi (immediately after coating), 4 hpi (co-culture for 4 h), or 12 hpi (co-culture for 12 h), and RNA was extracted for qRT-PCR. The gene changes in transcript abundance were normalized with RPKM. For qRT-PCR, the gene expression was normalized against that of β-tubulin gene in S. scleritorum. The qRT-PCR valued the gene expression at 0 hpi set as 1. (a) SsNEP1; (b) BcNEP1; (c) BcNEP2. The primers of qRT-PCR for SsNEP1, BcNEP1, and BcNEP2 were listed in Supplementary Table S7. [file Image_1.TIFF]
